# m6A‐Mediated Methylation Patterns and Their Association With Obstructive Sleep Apnea in Lung Adenocarcinoma

**DOI:** 10.1002/cnr2.70344

**Published:** 2025-09-08

**Authors:** Lei Xia, Zhuodong Chai, Xiaoying Wei, Yuan Wei, Daxiong Zeng, Junhong Jiang, Jing Li

**Affiliations:** ^1^ Department of Respiratory and Critical Care Medicine The Fourth Affiliated Hospital of Soochow University, Suzhou Dushu Lake Hospital, Medical Centre of Soochow University Suzhou Jiangsu China; ^2^ Department of Pharmaceutical Sciences Irma Lerma Rangel School of Pharmacy, Texas A&M University Texas USA; ^3^ Department of Respiratory and Critical Care Medicine First Affiliated Hospital of Soochow University Suzhou China

**Keywords:** immune infiltration, immunotherapy, lung adenocarcinoma, m6A, microenvironment, mutation burden, OSAHS, RNA‐seq

## Abstract

**Background:**

Epigenetic regulation significantly affects immune responses in lung adenocarcinoma (LUAD). However, the role of RNA N6‐methyladenosine (m6A) modification, especially in obstructive sleep apnea‐hypopnea syndrome (OSAHS) within LUAD, is not well understood.

**Methods:**

This study examined m6A modification patterns in 973 LUAD patients using 23 regulatory genes. Unsupervised clustering categorized patients by m6A profiles, quantified by an m6A score. Associations with clinical outcomes, including survival, immune infiltration, tumor microenvironment, OSAHS incidence, and drug sensitivity, were analyzed.

**Results:**

m6A regulator mutations were linked to LUAD, revealing two distinct modification patterns and three gene clusters, all tied to immune phenotypes and clinical outcomes. Higher m6A scores correlated with lower survival, more frequent OSAHS, and increased immune activity. Patients with high m6A scores were more sensitive to PD‐L1/PD‐1 inhibitors and Phenformin, while those with low scores responded better to Cisplatin, Epothilone B, and Talazoparib.

**Conclusion:**

Analyses confirmed m6A modification's role in LUAD progression, immune regulation, and outcomes. The m6A score is a valuable prognostic marker associated with survival, OSAHS incidence, and drug sensitivity. These findings highlight m6A's potential as a therapeutic target and biomarker, particularly for LUAD patients with OSAHS.

**Abbreviations**
CNV: copy number variation
EMT: epithelial‐mesenchymal transition
FPKM: fragments per kilobase
GEO: gene expression omnibus
GSEA: gene‐set enrichment analysis
GSVA: gene‐set variation analysis
IH: intermittent hypoxia
LUAD: lung adenocarcinoma
MDSC: myeloid‐derived suppressor cells
OS: overall survival
OSAHS: obstructive sleep apnea hypoventilation syndrome
PFS: progression‐free survival
TAM: tumor‐associated macrophages
TCGA: The Cancer Genome Atlas
TPM: transcripts per kilobase million
Treg: regulatory T cells

## 1 Introduction

Lung adenocarcinoma (LUAD) is the most common subtype of lung cancer, comprising over half of all cases globally. Enhanced screening technologies have improved early detection rates for LUAD through surveillance programs [1, 2]. Obstructive Sleep Apnea‐Hypopnea Syndrome (OSAHS) is a widespread sleep disorder caused by repeated airway blockages during sleep, leading to intermittent oxygen deprivation [3]. Lung cancer patients have a higher risk of OSAHS due to airway obstruction from cancer‐related structural changes [4]. OSAHS is also linked to various systemic health issues, such as hypertension and cardiovascular disease [5]. Research suggests a connection between OSAHS and lung cancer progression [6], as intermittent hypoxia (IH) may alter the tumor microenvironment and promote metastasis [7]. Chronic inflammation from OSAHS could further drive cancer development, highlighting its role beyond being an isolated health condition [8].

Epigenetic modifications like 5‐methylcytosine (m5C), N6‐methyladenosine (m6A), and N1‐methyladenosine (m1A) play key roles in biological regulation [9]. m6A methylation, a dynamic and reversible cellular process, is controlled by methyltransferases (“writers”), RNA‐binding proteins (“readers”), and demethylases (“erasers”) [10]. Major regulators include RBM15, METTL3, METTL14, ZC3H13, VIRMA (KIAA1429), WTAP, FTO, and ALKBH5 [11]. “Readers” like YTHDC1/2 and YTHDF1/2/3 recognize m6A marks, influencing RNA metabolism [12]. Disruptions in m6A regulatory factors are linked to various pathological conditions, including tumor progression and impaired cell self‐renewal [13, 14]. Understanding m6A's role in these processes is essential for exploring its regulatory impact [15, 16].

Previously, tumor development was mainly linked to genetic and epigenetic changes within cancer cells [17]. However, recent research emphasizes the tumor microenvironment (TME), consisting of both cancerous and stromal cells, as a key factor in tumor progression [18]. Aberrant m6A regulation is associated with immune‐cell infiltration in the TME. Normally, immune cells like CD8+ T cells and natural killer cells in the TME suppress tumor growth, but abnormal m6A modifications can weaken their surveillance abilities [19]. Therefore, evaluating TME infiltration patterns influenced by m6A regulators is crucial to understanding immune modulation within the TME.

OSAHS has been associated with LUAD progression; the specific epigenetic changes predisposing LUAD patients to OSAHS are still unclear. OSAHS is mainly driven by periodic airway collapse, systemic inflammation, and metabolic issues [20]. Repeated IH in OSAHS induces oxidative stress and chronic inflammation, promoting the secretion of factors like IL‐6 and TNF‐α. These factors worsen immune suppression in the tumor microenvironment and influence m6A modifications, affecting RNA fate and promoting cancer growth [21, 22, 23, 24]. Additionally, IH activates hypoxia‐inducible factor HIF‐1α, which modifies m6A methylation, altering tumor cell gene expression and enhancing their growth and immune evasion [25, 26, 27]. OSAHS is also linked to metabolic syndrome and insulin resistance, contributing to metabolic stress in the tumor microenvironment and LUAD progression [28, 29]. In obese patients, fat accumulation and limited diaphragm movement worsen airway collapse, creating a cycle between LUAD and OSAHS, with m6A methylation playing a central role.

Despite this established association, the precise epigenetic changes, especially those related to m6A, remain poorly defined. This study explores how m6A regulator‐mediated methylation patterns may explain the increased incidence of OSAHS in LUAD. By examining m6A methylation and its regulatory networks, we aim to identify epigenetic markers linked to OSAHS in LUAD. We analyzed genetic data from 973 LUAD samples across seven databases, focusing on m6A profiles and their relationship with TME immune‐cell infiltration. Our findings show that m6A modification patterns are linked to immune rejection, inflammation, and immunodeficiency in the TME, highlighting m6A's role in shaping the tumor's immune environment. To enhance clinical relevance, we developed a scoring system to quantify specific m6A patterns as potential biomarkers for OSAHS risk and LUAD prognosis. This approach may facilitate early diagnosis and personalized treatments, improving patient outcomes.

## 2 Material and Methods

### 2.1 Source and Preprocessing of Lung Adenocarcinoma Data

Gene expression datasets with clinical annotations were obtained from The Cancer Genome Atlas (TCGA) and the Gene Expression Omnibus (GEO). Patients without survival data were excluded. The study included cohorts from GSE3141, GSE37745, GSE19188, GSE30219, GSE50081, GSE8894, and TCGA‐LUAD (Table 1).

**TABLE 1:** Characteristics of the included TCGA and GEO datasets.

Database	ID	Number of cases	Analysis type	Platform
TCGA	LUAD	481	Array	TCGA
GEO	GSE19188	39	Array	GPL570
GEO	GSE30219	85	Array	GPL570
GEO	GSE3141	57	Array	GPL570
GEO	GSE37745	106	Array	GPL570
GEO	GSE50081	129	Array	GPL570
GEO	GSE8894	76	Array	GPL570

Abbreviations: GEO, Gene Expression Omnibus; LUAD, Lung Adenocarcinoma, TCGA, The Cancer Genome Atlas.

Raw data underwent preprocessing, including correction and normalization using the robust multi‐array averaging (RMA) algorithm via the “simpleaffy” and “affy” R packages. Principal component analysis (PCA) was conducted on the RNA‐seq data. Normalized expression matrices were retrieved from each platform.

TCGA gene expression data in fragments per kilobase million (FPKM) format were accessed through the Genomic Data Commons (GDC) using the “TCGA‐biolinks” R package and then converted to transcripts per kilobase million (TPM). Batch effects were corrected using the “ComBat” algorithm from the “sva” package. Somatic mutation data were also sourced from TCGA. Data processing was performed using R software (v4.3.1) [30].

### 2.2 Analysis of 23 m6A Regulators

We selected 23 m6A regulatory factors from the TCGA and GEO datasets, including 2 demethylases (“erasers” – FTO and ALKBH5), 8 methyltransferases (“writers” – METTL3, METTL14, METTL16, RBM15, RBM15B, WTAP, ZC3H13, and VIRMA), and 13 RNA‐binding proteins (“readers” – YTHDC2, LRPPRC, YTHDF2, YTHDF3, YTHDF1, YTHDC1, IGF2BP2, HNRNPC, IGF2BP3, IGF2BP1, FMR1, HNRNPA2B1, and RBMX). Agglomerative clustering analysis was performed on these factors to identify distinct m6A modification patterns. The reliability and stability of clustering were validated using a classification algorithm, and the “ConsensusClusterPlus” package in R, with 2000 iterations, was applied to ensure consistency [31].

### 2.3 Gene‐Set Variation Analysis and Immune‐Cell Infiltration Assessment

To investigate biological processes linked to m6A modification patterns, we used the “GSVA” package for Gene‐Set Variation Analysis (GSVA), a non‐parametric, unsupervised method to assess pathway activity variations [32]. The “c2.cp.kegg.v6.2” gene set from the Molecular Signatures Database (MSigDB) was used for pathway analysis, with gene symbols for pathway loading. Statistical significance was set at *p* < 0.05. Functional annotation of m6A‐related genes was performed using the “clusterProfiler” R package, applying a false discovery rate (FDR) threshold of less than 0.05.

To assess immune‐cell infiltration in the tumor microenvironment (TME), we employed single‐sample Gene‐Set Enrichment Analysis (ssGSEA). Immune subtypes, including regulatory T cells, natural killer T cells, macrophages, activated CD8 T cells, and immature dendritic cells, were characterized based on Charoentong et al.'s classification [33, 34]. Enrichment scores from ssGSEA were calculated to measure the relative abundance of these cell types in the TME.

### 2.4 Assessing Differentially Expressed Genes (DEGs) and Pathway Analysis

We evaluated the relationship between gene expression and m6A modifications by examining the expression of 23 m6A regulatory factors. Patients were categorized into three m6A phenotypes (gene clusters), and differential gene expression analysis was conducted using the “limma” package, identifying significant changes with an adjusted *p*‐value < 0.01.

To identify DEGs across groups, we applied the “limma” package with criteria of log2 |fold change| > 1 and a FDR < 0.05. Further analysis of DEGs' biological functions and signaling pathways was performed using “clusterProfiler,” “org.Hs.eg.db,” and “enrichplot” R packages, focusing on Gene Ontology (GO) terms and Kyoto Encyclopedia of Genes and Genomes (KEGG) pathways to uncover their molecular mechanisms and implications.

### 2.5 Gene Signature of m6A and Its Association With Biological Processes

To characterize m6A modification patterns in LUAD, we developed a scoring system to assess these patterns. DEGs from m6A clusters were normalized, and overlapping genes were identified and clustered using an unsupervised approach. The consensus clustering algorithm defined the optimal gene clusters, followed by a univariate Cox regression model for prognostic analysis. PCA was used to create m6A‐associated gene signatures based on principal components 1 and 2, defining the m6A score similar to the Genomic Grade Index [35, 36]. The m6A score for each sample *i* was defined as:

$$m6A\;\text{Score}i=\sum k=1^{2}\sum_{j=1}^{g}{\mathrm{PC}}_{k,\mathrm{ij}}$$

where PC (kij) denotes the contribution of gene *j* to the *k*‐th principal component in sample *i*. This vector‐projection strategy, borrowed from the Genomic Grade Index, captures the coordinated variability of the signature genes while remaining insensitive to gene‐set size. As an alternative that separates protective and risk genes, the score can be formulated as

$$m6A\;\text{Score}i=\left(\sum j\in G_{\left(+\right)}\mathrm{PC}1\mathrm{ij}\right)-\left(\sum j\in G_{\left(-\right)}\mathrm{PC}1_{\mathrm{ij}}\right)$$

where G (+) and G (−) are genes with positive or negative Cox coefficients, respectively. Bootstrap validation (1000 iterations, 70/30 train–test split) showed the first formula yielded a higher cumulative C‐index (0.712 vs. 0.691); hence, it was adopted for all downstream analyses.

We then explored the relationship between m6A gene signatures and biological processes, including antigen presentation, immune checkpoints, fibroblast TGF‐β response, CD8 T‐cell signatures, epithelial‐mesenchymal transition, and DNA repair, based on the clusters identified by Mariathasan et al. [37, 38].

### 2.6 Assessment of Drug Sensitivity

The clinical relevance of m6A genes in LUAD treatment was evaluated by calculating the half‐maximal inhibitory concentration (IC50) for 138 drugs using the “pRRophetic” R package and associated tools (“ridge,” “car,” “preprocessCore,” “genefilter,” “sva”). Sensitivity differences between high‐ and low‐m6A score groups were assessed with the Wilcoxon signed‐rank test, and results were visualized using “ggplot2.”

### 2.7 Statistical Analysis

We used Spearman's rank and distance correlation analyses to examine the relationship between m6A regulators and immune‐cell infiltration in the TME. Differences across groups were analyzed using one‐way ANOVA and the Kruskal‐Wallis test [39]. Correlations between the m6A score and immune‐cell infiltration were assessed with Spearman's test, and only associations meeting |ρ| ≥ 0.20 together with *p* < 0.05 were deemed biologically relevant and plotted.

The “survminer” R package was used to determine cutpoints for stratifying patients into high and low‐m6A score groups. Independent prognostic factors were identified using multivariate Cox regression models. The m6A score's sensitivity and specificity were assessed with receiver operating characteristic (ROC) curves, and the area under the curve (AUC) was calculated via the “pROC” package. We visualized mutations in high and low‐m6A score subtypes from the TCGA‐LUAD cohort using the “maftools” package, while variations in m6A regulators across chromosome pairs were illustrated with “RCircos” [40]. All tests were two‐sided, with significance set at *p* < 0.05.

## 3 Results

### 3.1 Genetic Changes of m6A Regulators in Lung Adenocarcinoma

**FIGURE 1:**
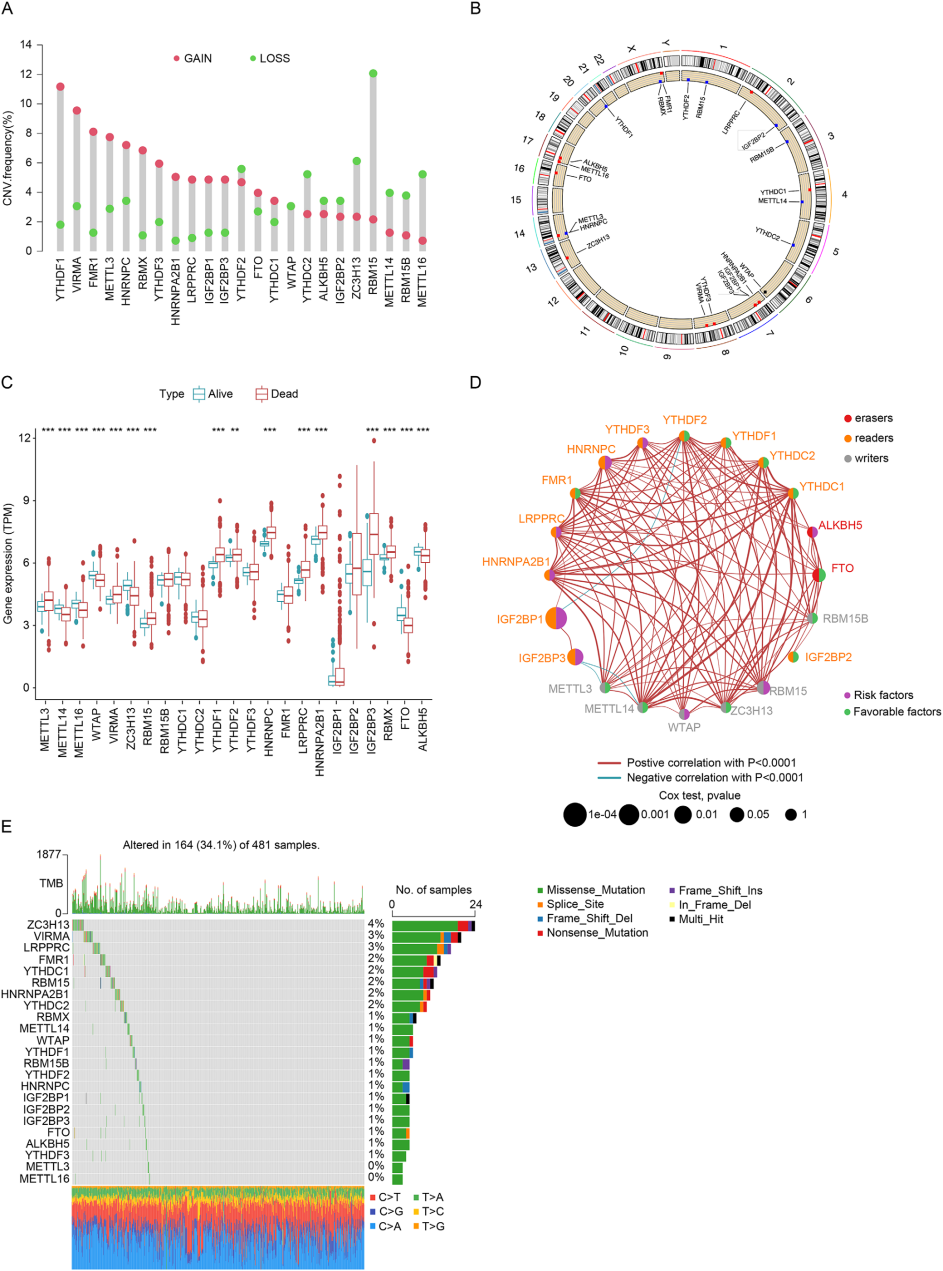
m6A regulators in LUAD. (A) CNV frequencies of m6A regulators in the TCGA dataset; green dots show deletions, red dots show amplifications. (B) Chromosomal locations of CNV changes. (C) Expression levels (TPM) of 18 m6A regulators in GEO and TCGA datasets; survival data from 973 samples is color‐coded (blue: Survival, red: Death). (D) Interaction network of m6A regulators; risk (purple dots) and protective factors (green dots). (E) Mutation profiles of m6A‐related genes.

In LUAD, 23 m6A regulators were identified, including 8 methyltransferases, 2 demethylases, and 13 RNA‐binding proteins. Copy number variation (CNV) analysis revealed widespread alterations, with most regulators showing gains; however, RBM15 had a higher frequency of CNV loss (Figure 1A). The chromosomal distribution of these CNV variations is shown in Figure 1B.

To understand how genetic variations affect m6A regulator expression in LUAD, we compared mRNA levels between tumor and normal tissues (Figure 1C). Most regulators, such as METTL3, YTHDF1, IGF2BP3, and RBMX, were upregulated in tumor tissues, while METTL14 and ZC3H13 were downregulated. Overexpression may be linked to CNV amplification, though not all genes followed this pattern. These results highlight the genetic and expression heterogeneity of m6A regulators and their role in LUAD progression.

### 3.2 Interaction Network and Prognostic Implications of m6A Regulators in Lung Adenocarcinoma

Data from TCGA‐LUAD and GEO datasets (GSE19188, GSE3141, GSE37745, GSE30219, GSE50081, GSE8894) were used to construct an m6A regulatory network, examining interactions and prognostic significance in LUAD (Figure 1D). Significant correlations were observed among 20 m6A regulators, forming distinct clusters of “writers,” “erasers,” and “readers.” Positive correlations (red lines) indicated coordinated regulation, while negative correlations (blue lines) represented inverse patterns. Some regulators were identified as either risk (purple nodes) or favorable factors (green nodes) for patient survival. METTL16, VIRMA, and RMBX showed no notable associations and were excluded. These interactions highlight the dynamic role of m6A factors in shaping modification profiles.

Mutation analysis of 481 LUAD samples from the TCGA showed that 34.10% (164 samples) had mutations in at least one m6A‐related gene, indicating the complexity of genetic alterations in this pathway. These mutations could disrupt m6A methylation, influencing tumor progression through altered RNA processing and expression.

### 3.3 Characterization of TME Patterns Based on m6A Modifications

**FIGURE 2:**
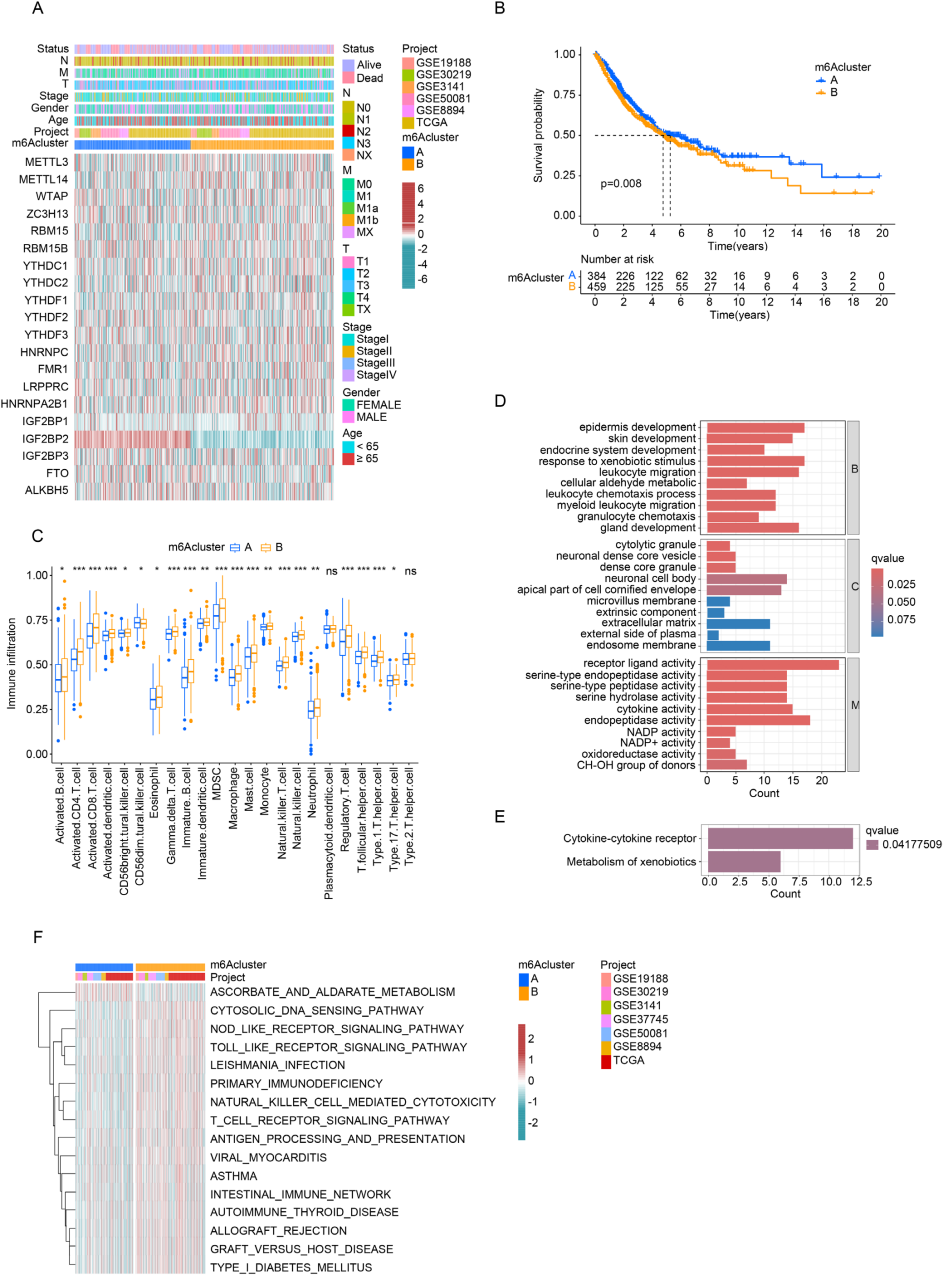
TME cell characteristics under m6A patterns. (A) Heatmap of 18 m6A regulators; low expression (blue), high expression (red). (B) Survival analysis of 973 LUAD patients. (C) TME cell abundance under different m6A patterns; statistical significance marked (*p < 0.05; **p < 0.01; ***p < 0.001). (D) GO enrichment; bar length indicates gene count. (E) KEGG enrichment; bar length reflects gene numbers. (F) Heatmap of differential pathways between m6A clusters.

Unsupervised clustering of m6A modifications in LUAD samples identified two distinct clusters, m6Acluster‐A and m6Acluster‐B (Figure 2A). m6Acluster‐A showed elevated expression of IGF2BP2, FTO, and ALKBH5, while m6Acluster‐B had higher levels of RBM15, METTL14, and YTHDC2. Patients in m6Acluster‐B exhibited lower overall survival rates compared to those in m6Acluster‐A, indicating a significant association between m6A patterns and prognosis (Figure 2B).

Further analysis of the tumor microenvironment (TME) revealed that m6Acluster‐B was enriched with innate immune cells, including macrophages, regulatory T cells, mast cells, natural killer cells, and MDSCs (Figure 2C). KEGG and GO analyses of DEGs between the clusters indicated involvement in processes such as leukocyte migration, granulocyte chemotaxis, cytokine activity, and cytokine–cytokine receptor signaling (Figure 2D,E). GSVA showed that m6Acluster‐B was significantly enriched in oncogenic pathways like the NOD‐like and Toll‐like receptor signaling pathways (Figure 2F). These findings underscore the crucial role of m6A modifications in influencing immune‐cell migration and oncogenic signaling in the TME, potentially driving LUAD progression.

### 3.4 Transcriptome and Clinical Profiles of m6A‐Associated Phenotypes

**FIGURE 3:**
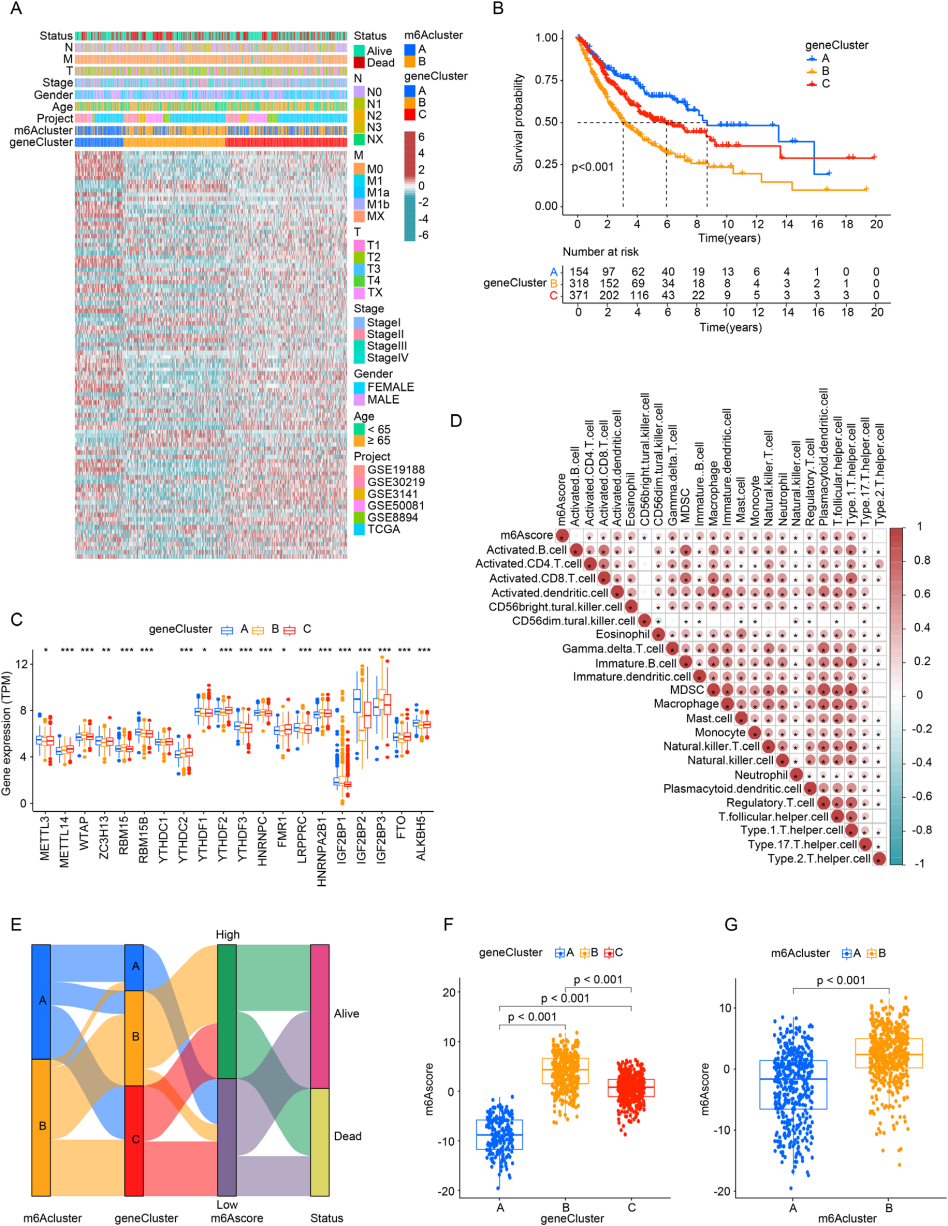
m6A signatures. (A) Clustering of 973 patients into m6A gene clusters A, B, and C. (B) Kaplan–Meier curves showing the link between m6A‐modified phenotype and overall survival. (C) Expression profiles (TPM) of 20 m6A regulators (*p < 0.05; **p < 0.01; ***p < 0.001). (D) Correlation between m6A scores and immune infiltration. (E) Alluvial plot of m6A clusters, gene clusters, m6A scores, and survival states. (F) m6A score differences among gene clusters (*p* < 0.001). (G) m6A score variations in m6A patterns.

Unsupervised clustering of LUAD patient genes revealed three genomic types: gene clusters A, B, and C (Figure 3A). Tumors in cluster B were associated with more severe characteristics, such as higher T stage and increased lymph node involvement, while clusters A and C indicated less aggressive features. Patients in clusters A and C were older (Median [IQR]: 57[45–67] vs. 42 [35, 36, 37, 38, 39, 40, 41, 42, 43, 44, 45, 46, 47, 48, 49, 50, 51, 52, 53, 54]) and had higher mortality rates (75.2% vs. 53.1%).

Among the 973 patients, 187 fell into cluster A, which correlated with a favorable prognosis. In contrast, clusters B and C (381 and 405 patients, respectively) were linked to poorer outcomes (Figure 3B). Survival differences between clusters were significant (*p* < 0.001), demonstrating the classification's ability to distinguish patient prognoses.

RNA‐seq analysis showed significant variations in m6A‐modified gene expression across clusters, consistent with expected m6A methylation patterns (Figure 3C). Gene clustering proved more predictive than m6A clustering, highlighting the substantial influence of m6A regulators on shaping genomic expression.

### 3.5 Gene Signature of m6A Modification

To assess the role of m6A modifications in LUAD, we developed an m6A score using PCA of m6A‐related gene expression. This score quantifies overall m6A modification patterns for each patient by focusing on the most correlated gene sets, minimizing unrelated noise. The m6A score was then correlated with immune‐cell traits, revealing positive associations with activated dendritic cells, gamma delta T cells, Type I helper cells, and natural killer T cells (Figure 3D). These findings suggest that m6A modifications may impact the tumor immune microenvironment, influencing immune responses in LUAD.

To explore the prognostic significance, we analyzed m6A clusters, gene clusters, m6A scores, and survival outcomes. An alluvial plot (Figure 3E) visualized transitions between clusters and survival states. Significant differences in m6A score were found across gene clusters, with cluster A having the lowest median score and cluster B the highest (Figure 3F). This pattern aligns with clinical outcomes (Figure 3B) and was also observed between m6A cluster A and B (Figure 3G). These results validate the clustering method and highlight the prognostic value of the m6A score in predicting patient outcomes.

### 3.6 Impact of m6A score and Tumor Mutation Burden on Survival and OSHAS

**FIGURE 4:**
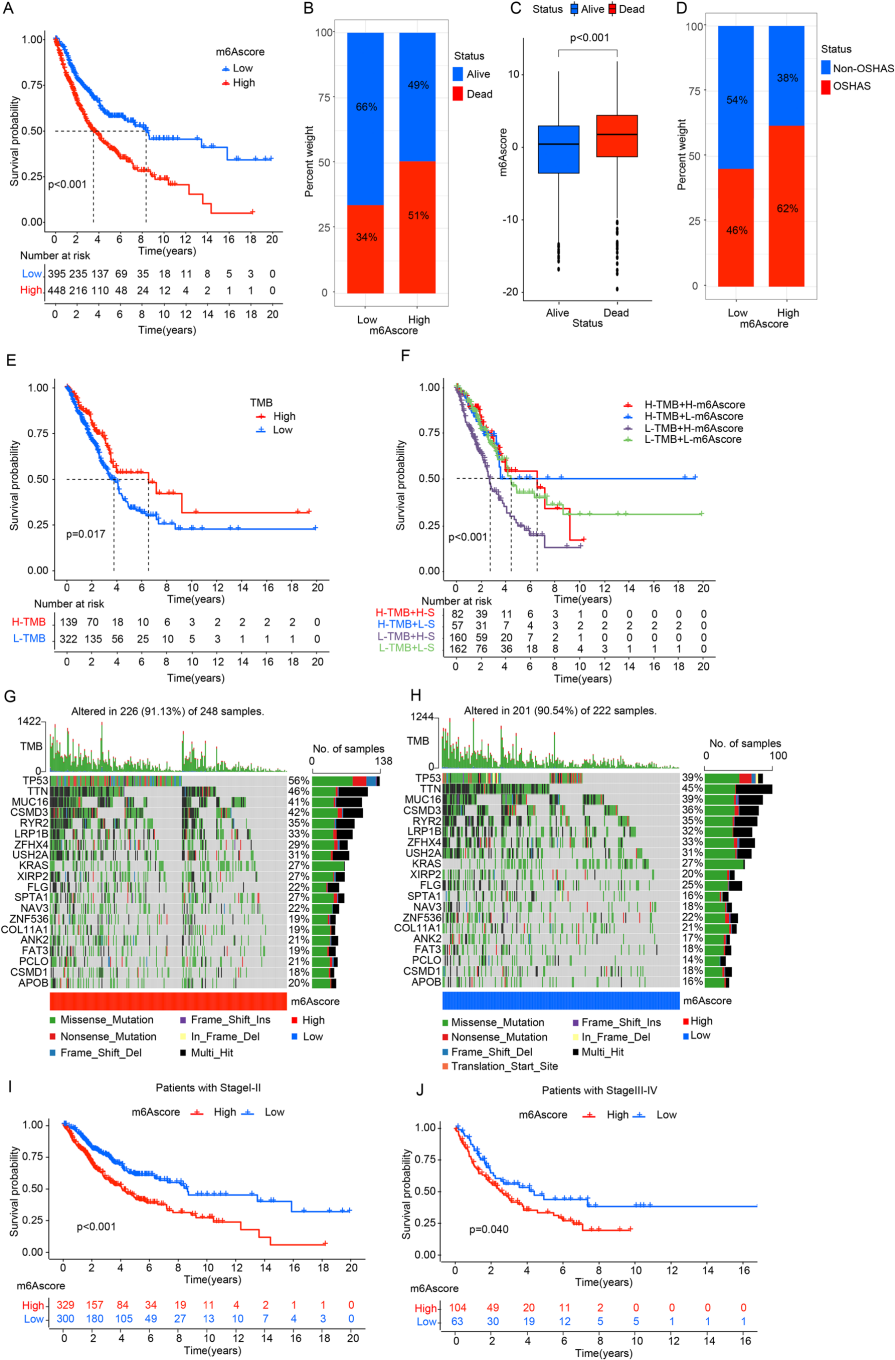
m6A modification, tumor mutations, and survival. (A) Kaplan–Meier survival analysis for low (395) and high (448) m6A score groups (*p* < 0.001). (B) m6A score and survival rate correlation. (C) Boxplot of m6A score with survival/death status. (D) m6A score correlation with OSAHS incidence. (E) Survival based on TMB using Kaplan–Meier curves (*p* = 0.017). (F) Survival by m6A score and TMB (*p* < 0.001). (G‐H) Mutation profiles for high and low m6A score groups. (I–J) Survival stratification by m6A score in stages I‐II and III‐IV (*p* < 0.001).

Using the “survminer” package, we divided patients into m6A score groups based on a cutoff of −7.31. Higher m6A scores were linked to worse prognosis, with lower scores showing a twofold increase in 5‐year survival (57.9% vs. 26.1%) (Figure 4A). Higher m6A scores also correlated with increased mortality rates (Figure 4B,C). Additionally, 62% of patients with elevated m6A scores had OSAHS, compared to 46% in the low‐m6A score group, indicating a potential link between m6A methylation and OSAHS incidence in LUAD (Figure 4D).

Patients were further stratified by tumor mutation burden (TMB). Those with lower TMB had a poorer prognosis than those with high TMB (Figure 4E). Combining m6A score and TMB stratification showed that patients with low TMB and high‐m6A score had the worst outcomes, while high TMB and low‐m6A score indicated the best prognosis (Figure 4F).

Mutation profiles in different m6A score groups (Figure 4G,H) revealed that high‐m6A score patients had a significantly higher TMB, with common mutations in genes like TP53, CSMD3, PCLO, XIRP2, and ANK2. Spearman correlation showed a significant relationship between TMB and m6A score (*r* = 0.92, *p* = 0.023), suggesting an interplay between m6A modifications and TMB.

**FIGURE 5:**
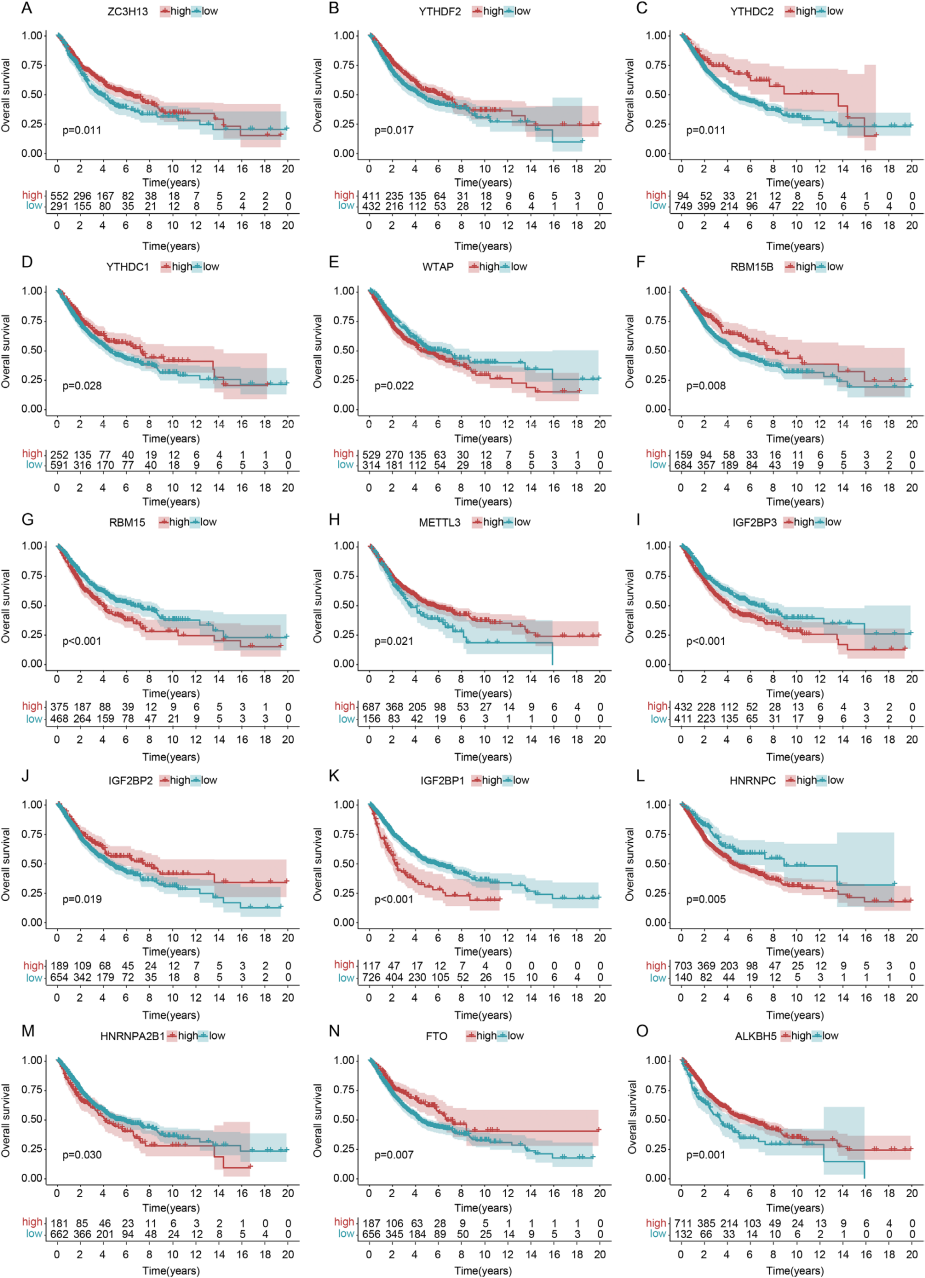
Kaplan–Meier curves for m6A genes. High expression of ZC3H13 (A), YTHDF2 (B), YTHDC2 (C), YTHDC1 (D), and others linked to better prognosis, while lower expression of WTAP (E), RBM15 (G), IGF2BP1 (K), and others is favorable (*p* < 0.05).

Stratifying patients by LUAD stage reinforced these findings, with lower m6A scores consistently indicating better survival, especially in early stages (*p* < 0.001) (Figure 4I,J). Further analysis of m6A‐related genes identified that higher expression of genes like ZC3H13, YTHDF2, YTHDC2, and YTHDC1 correlated with favorable prognosis, while genes such as WTAP, IGF2BP1, and HNRNPC were associated with better outcomes when expressed at lower levels (Figure 5).

### 3.7 Analysis of Treatment Targets and Drug Sensitivity

Given the impact of m6A scores on LUAD prognosis, we analyzed potential therapeutic targets. Patients with high m6A scores showed elevated PD‐L1 levels, suggesting increased sensitivity to PD‐L1‐targeted therapies (Figure S1).

**FIGURE 6:**
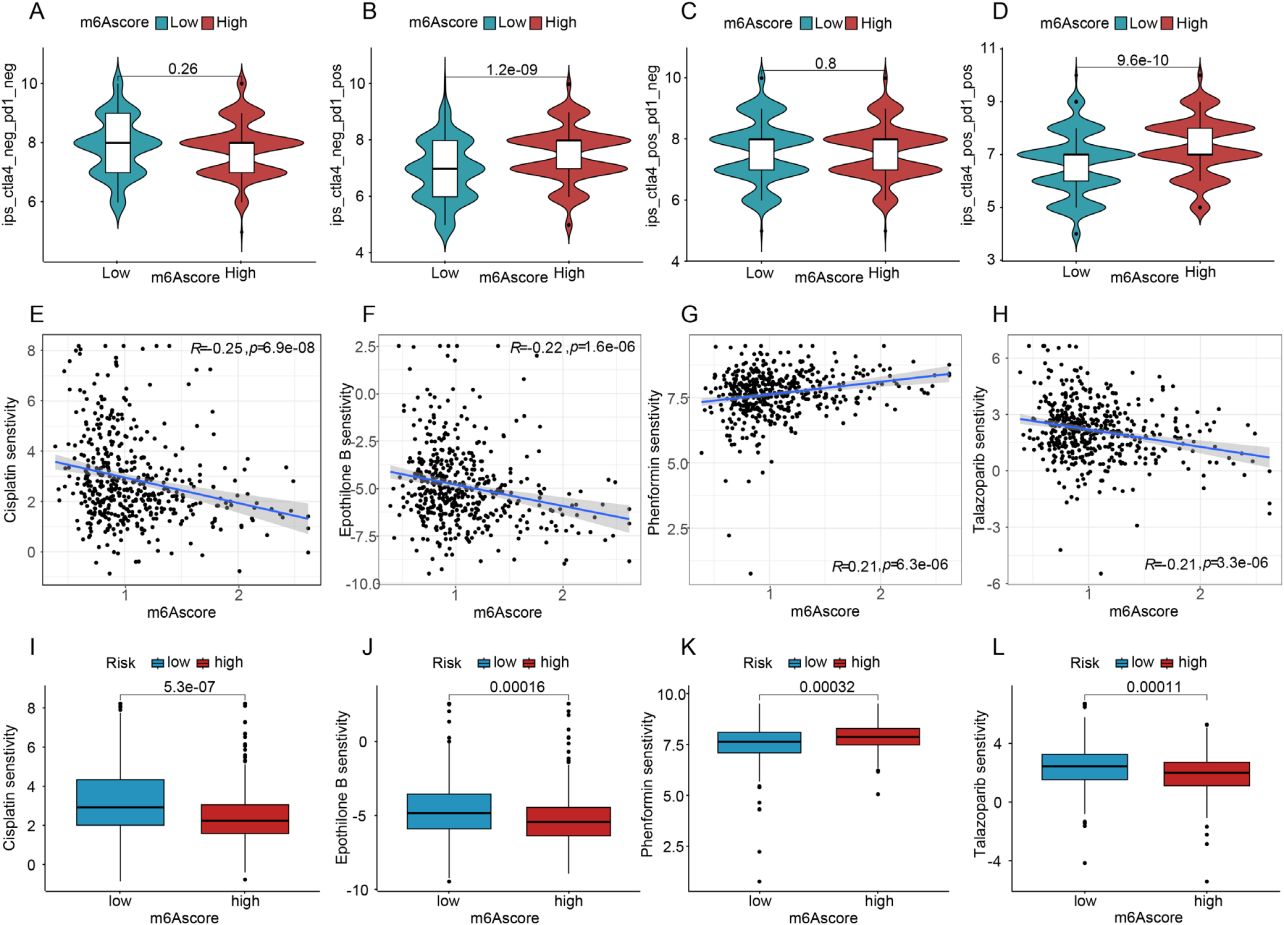
Treatment and drug sensitivity. (A–D) PD‐1 monotherapy/CTAL4 treatment in m6A score groups (TCGA data). (E–H) Scatter plots of drug sensitivity (Cisplatin, Phenformin, Epothilone B, Talazoparib) vs. m6A score. (I–L) Drug sensitivity in different m6A score groups.

Immunotherapy response analysis from the TCGA database revealed that patients in the high‐m6A score group responded better to PD‐1 monotherapy and combination therapy with CTLA‐4 (Figure 6A–D). These patients exhibited stronger immune responses in PD‐1‐positive cases, indicating that m6A score could serve as a predictive marker for immunotherapy efficacy. PD‐1‐negative cases showed no significant differences.

Further drug sensitivity analysis using the “pRRophetic” package (Figure 6E–H) found that Phenformin was more effective in patients with high m6A scores, while Cisplatin, Epothilone B, and Talazoparib were more effective in those with low m6A scores. Sensitivity values across different m6A score groups (Figure 6I–L) confirmed the varying drug efficacy based on m6A score levels.

## 4 Discussion

m6A is the most common mRNA modification, involving methyl group addition by methyltransferases (e.g., METTL3) and removal by demethylases (e.g., FTO, ALKBH5). This dynamic process is crucial for maintaining m6A balance, affecting RNA splicing, transport, translation, and degradation. Disruption in m6A regulation can lead to RNA metabolism issues [41, 42].

Our analysis identified 23 m6A regulators in LUAD, with CNV alterations significantly impacting their expression. Amplified regulators like METTL3, YTHDF1, and IGF2BP3 showed higher expression in tumors, while METTL14 and ZC3H13 were downregulated, indicating a complex link between CNVs and mRNA expression. These findings align with previous studies, highlighting the role of m6A genes in lung cancer and other tumors [43, 44, 45, 46]. Additionally, we characterized the interaction network of m6A regulators, uncovering key risk and protective factors that influence LUAD progression.

We identified two distinct m6A modification clusters with different immune infiltration patterns and survival outcomes. m6Acluster‐B, enriched with macrophages, Tregs, and MDSCs, was linked to a poorer prognosis, while m6Acluster‐A, showing upregulation of FTO and IGF2BP2, correlated with better outcomes [47]. Enrichment analysis indicated that m6A plays a regulatory role in immune pathways and the tumor microenvironment. M2‐type TAMs, Tregs, and MDSCs promote tumor growth and immune evasion by secreting factors like IL‐10, TGF‐β, VEGF, and MMP9 [48]. Our study also identified three gene clusters, further refining LUAD heterogeneity. Gene clusters A and C were linked to older patients, advanced tumors, and poorer survival, highlighting the significance of m6A modifications in defining genomic subtypes. Notably, our work is among the first to establish a link between OSAHS and LUAD through m6A methylation, showing how OSAHS‐induced hypoxia and inflammation may promote immune evasion in the tumor microenvironment.

The m6A score was shown to be a strong prognostic marker, significantly correlating with immune infiltration in the tumor microenvironment [45, 49, 50, 51]. A lower‐m6A score was associated with better survival. Combining the m6A score with TMB allows for more precise patient stratification, with those having low TMB and high‐m6A score showing the worst outcomes. Notably, while high TMB is generally linked to better survival [52, 53], our analysis revealed that the m6A score had a more significant impact on prognosis (*p* < 0.001) compared to TMB (*p* = 0.017). High‐m6A score clusters exhibited mutations in genes like TP53, CSMD3, and ANK2, which are associated with adverse outcomes in various cancers. Additionally, the m6A score holds greater prognostic value for early‐stage patients (Stage I‐II).

Although our study provides insights into m6A methylation's role in LUAD and immune infiltration, it has limitations. Using bulk RNA sequencing data obscures cell‐type‐specific m6A modifications. Single‐cell RNA sequencing or spatial transcriptomics could better clarify m6A's differential regulation in various cell populations. Furthermore, the clinical application of m6A as a biomarker faces challenges due to the complexity of detecting specific m6A modifications. Current methods, like MeRIP‐seq, have low resolution, necessitating the development of more refined techniques.

Our study also identified a potential link between m6A methylation and OSAHS in LUAD patients. OSAHS was more common in the high‐m6A score group, indicating m6A's role in OSAHS pathogenesis. IH from OSAHS promotes a pro‐tumorigenic environment through inflammation and oxidative stress, which can disrupt m6A modifications, creating a feedback loop that accelerates cancer progression. Understanding the interplay between OSAHS, m6A methylation, and lung cancer could lead to targeted therapies addressing both epigenetic and physiological aspects of these conditions.

IH, the hallmark of OSAHS, induces recurrent surges of reactive oxygen species that activate NF‐κB, HIF‐1α, and other redox‐sensitive pathways, driving systemic oxidative stress, endothelial dysfunction, and DNA damage, all of which create a microenvironment conducive to tumor initiation and progression [54]. Emerging evidence further shows that the cyclical desaturations, sympathetic overdrive, and sleep fragmentation seen in OSAHS amplify inflammation and blunt antitumor immune surveillance, offering a mechanistic explanation for the epidemiologic link between OSAHS severity and poorer oncologic outcomes [55]. Importantly, interventions that restore upper‐airway patency—ranging from continuous positive airway pressure to phenotype‐guided surgical approaches—have been shown to curb oxidative‐stress biomarkers, suggesting they may also mitigate the oncogenic risk associated with OSAHS [56].

The interplay between OSAHS and LUAD affects systemic inflammation, metabolic dysregulation, and airway function. As LUAD progresses, tumor growth can compress nearby airways, exacerbating OSAHS symptoms such as apnea, daytime sleepiness, and hypoxia. This compression increases the risk of upper‐airway obstruction and leads to more IH. Additionally, tumor cells release abnormal metabolic factors and inflammatory mediators, inducing paraneoplastic syndrome, which disrupts pulmonary tissue function and causes metabolic and endocrine disturbances. Chronic inflammation and metabolic dysregulation weaken immune function, enhancing tumor invasiveness and metastasis.

Our findings suggest that aberrant m6A regulation is key in the interaction between OSAHS and LUAD, particularly in modulating the tumor microenvironment and immune infiltration. Investigating how m6A mechanisms influence hypoxia‐induced immune evasion may identify new therapeutic targets for LUAD patients with OSAHS. Although precise pathways remain unclear, m6A modifications may link the immune and metabolic dysregulation observed in both conditions. Further mechanistic studies are needed to explore the direct impact of m6A on the co‐occurrence of OSAHS and LUAD, potentially guiding individualized treatment strategies.

In lung cancer tissues, elevated expression of FTO and ALKBH5 promotes tumor cell proliferation, with m6A modifications playing a crucial role [57, 58]. These demethylases regulate lung cancer progression [59] and our study found that loss of METTL3 and FTO is linked to worse prognoses in LUAD [60]. Despite advancements, m6A‐targeted therapies remain unavailable for clinical use. Some small‐molecule inhibitors targeting FTO and ALKBH5 show promise in preclinical studies but are not yet clinically implemented. Notably, downregulation of FTO worsens outcomes in bladder and prostate cancers, reflecting the complexity of tumor development and underscoring the need for personalized treatment based on m6A status [61, 62].

Future research should focus on the m6A‐OSAHS‐LUAD connection, exploring m6A modifications as biomarkers and therapeutic targets. Assessing whether m6A‐targeted therapies can alleviate both OSAHS symptoms and LUAD progression could reveal new treatment avenues. Combining m6A inhibitors with therapies to reduce hypoxia‐induced stress, like immune checkpoint inhibitors, may improve outcomes in patients with co‐occurring LUAD and OSAHS.

We also identified that patients with high m6A scores exhibit increased PD‐L1 expression, suggesting a heightened sensitivity to PD‐L1/PD‐1 inhibitors, consistent with findings in breast cancer [63]. Current m6A demethylase drugs, such as FTO inhibitors like FB23, rhein, and 44/ZLD115, show potential, particularly 44/ZLD115's potent anti‐leukemia effects [64, 65]. Additionally, chemotherapy efficacy varies with m6A score levels, indicating that m6A alterations could predict responses to immunotherapy and chemotherapy in LUAD. Overall, our findings highlight the role of m6A regulation in LUAD, emphasizing its importance in prognosis prediction and therapeutic strategies. Future studies should delve into the functional mechanisms of m6A modifications as targets for new therapies.

## 5 Conclusions

Our study revealed significant CNV alterations in 23 m6A regulators in LUAD, highlighting their role in shaping the tumor microenvironment, particularly in immune‐cell infiltration and survival outcomes. Unsupervised clustering identified distinct m6A‐modified genomic types with clinical relevance.

Notably, we identified a potential interaction between OSAHS and LUAD via m6A methylation, with higher m6A scores linked to increased OSAHS risk. This novel connection underscores the potential of m6A as a therapeutic target, suggesting new avenues for personalized treatments.

Additionally, the m6A score emerged as a potential biomarker, correlating with worse survival and increased sensitivity to PD‐L1/PD‐1 inhibitors. Patients with higher m6A scores may benefit from PD‐L1/PD‐1‐targeted immunotherapy. Overall, our findings establish m6A methylation as a key factor in LUAD progression and immune modulation, offering opportunities for tailored treatment, especially for those with co‐occurring OSAHS.

## Availability of Data and Materials

Data and materials used in this study are available upon request.

## Author Contributions

Lei Xia, Zhuodong Chai, Xiaoying Wei, and Yuan Wei conducted data analysis and manuscript drafting. Jing Li contributed to data analysis. Daxiong Zeng and Junhong Jiang supervised and revised the manuscript.

## Ethics Statement

The authors have nothing to report.

## Consent

The authors have nothing to report.

## Conflicts of Interest

The authors declare no conflicts of interest.

## Supporting information


**Figure S1:** Scatter plot of PD‐L1 expression vs. m6A score in LUAD patients.

## Data Availability

The data that support the findings of this study are available on request from the corresponding author. The data are not publicly available due to privacy or ethical restrictions.
