# Supplementary figures and images for: m6A‐Mediated Methylation Patterns and Their Association With Obstructive Sleep Apnea in Lung Adenocarcinoma
Source: Cancer Rep (Hoboken). 2025 Sep 8;8(9):e70344. doi: 10.1002/cnr2.70344 (PMC12415354; doi:10.1002/cnr2.70344)

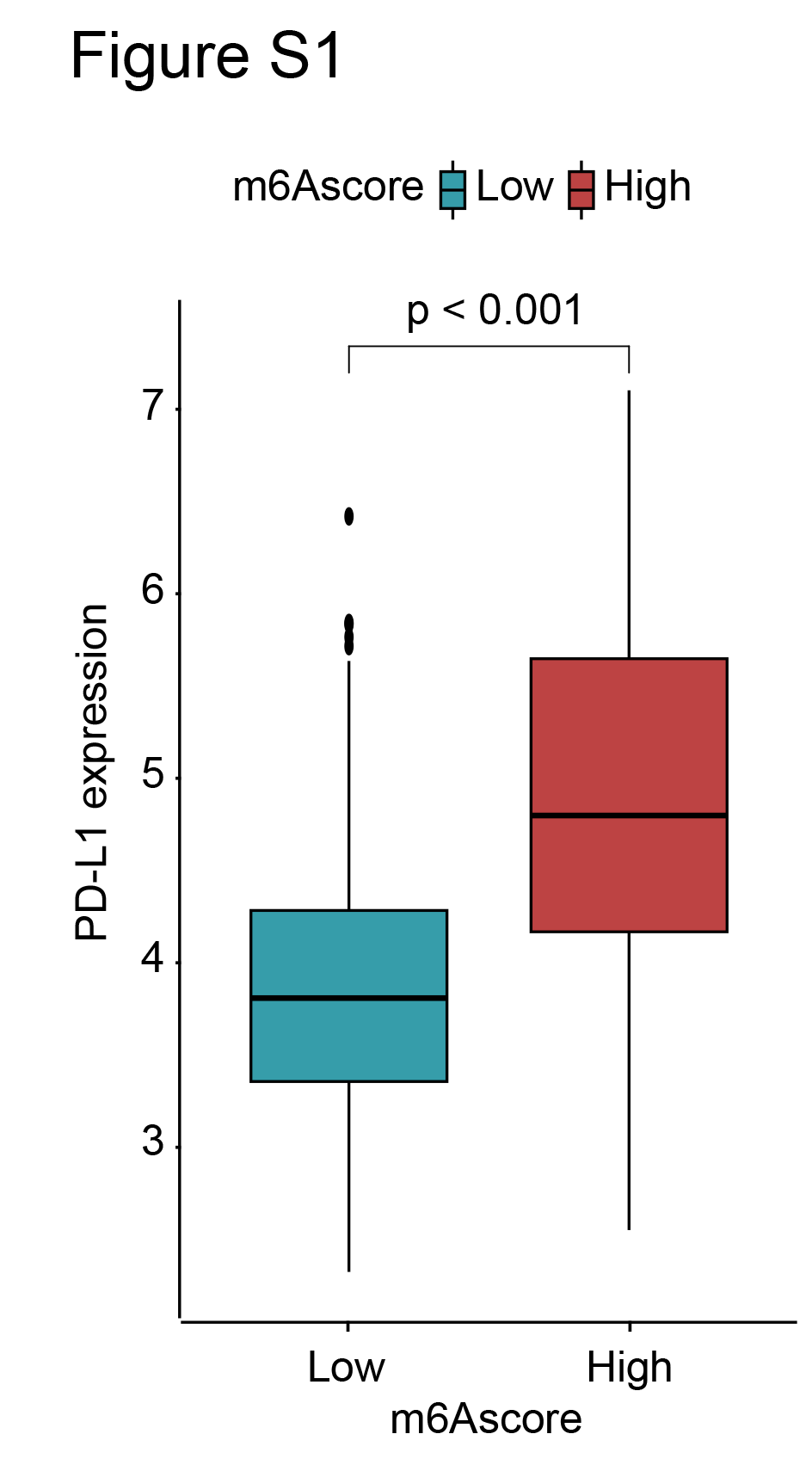

Supplement: Supplementary file 1 — Figure S1: Scatter plot of PD‐L1 expression vs. m6A score in LUAD patients. [file CNR2-8-e70344-s001.tif]
